# Development and validation of a risk prediction model for multiple organ dysfunction syndrome secondary to severe heat stroke based on immediate assessment indicators on ICU admission

**DOI:** 10.3389/fmed.2024.1481097

**Published:** 2024-12-24

**Authors:** Entong Ren, Hao Chen, Chenjiao Guo, Yuanyuan Peng, Li Tian, Lulu Yan, Huasheng Tong, Anwei Liu, Weihua Li

**Affiliations:** ^1^Health Science Center, Yangtze University, Jingzhou, Hubei, China; ^2^Department of Critical Care Medicine, General Hospital of Southern Theatre Command of PLA, Guangzhou, Guangdong, China; ^3^Department of Medicine, Guangdong Pharmaceutical University, Guangzhou, Guangdong, China; ^4^Department of Nursing, General Hospital of Southern Theatre Command of PLA, Guangzhou, Guangdong, China

**Keywords:** severe heat stroke, multiple organ dysfunction syndrome, prediction model, assessment indicators, ICU admission

## Abstract

**Introduction:**

Early prediction of multiple organ dysfunction syndrome (MODS) secondary to severe heat stroke (SHS) is crucial for improving patient outcomes. This study aims to develop and validate a risk prediction model for those patients based on immediate assessment indicators on ICU admission.

**Methods:**

Two hundred eighty-four cases with SHS in our hospital between July 2009 and April 2024 were retrospectively reviewed, and categorized into non-MODS and MODS groups. Logistic regression analyses were performed to identify risk factors for MODS, and then to construct a risk prediction model, which was visualized by a nomogram. The predictive performance of the model was evaluated using the area under the receiver operating characteristic curve (AUC), Hosmer-Lemeshow (HL) test, calibration curve, and decision curve analysis (DCA). Finally, the AUCs of the prediction model was compared with other scoring systems.

**Results:**

Acute gastrointestinal injury (AGI), heart rate (HR) >100 bpm, a decreased Glasgow Coma Scale (GCS) score, and elevated total bilirubin (TBil) within the first 24 h of ICU admission are identified as independent risk factors for the development of MODS in SHS patients. The model demonstrated good discriminative ability, and the AUC was 0.910 (95% *CI*: 0.856–0.965). Applying the predictive model to the internal validation dataset demonstrated good discrimination with an AUC of 0.933 (95% *CI*: 0.880–0.985) and good fit and calibration. The DCA of this model showed a superior clinical net benefit.

**Discussion:**

The risk prediction model based on AGI, HR, GCS, and TBil shows robust predictive performance and clinical utility, which could serve as a reference for assessing and screening the risk of MODS in SHS patients.

## 1 Introduction

Heat stroke is an acute disorder characterized by disturbances in the central nervous system and cardiovascular system resulting from imbalances in heat regulation or water and salt metabolism within a high-temperature environment. With ongoing climate warming and the increasing frequency of extreme heat events, the global incidence and mortality rates of heat stroke are increasing annually (1). By 2050 and 2080, the mortality rates are expected to increase 257 and 535%, respectively (2). Severe Heat stroke (SHS), the most critical type of heat stroke, typically manifests as a core body temperature (BT) exceeding 40°C, neurological dysfunctions such as seizures and coma, and damage to multiple organs, including the liver, kidney, intestine, lungs, coagulation, and skeletal muscle (3). SHS poses significant health risks and its incidence is projected to continue increasing, as highlighted in the China Population Health and Climate Change Report (4). Multiple organ dysfunction syndrome (MODS) is the most severe complication of SHS and is the primary cause of mortality in these patients (5). MODS significantly increases the risk of various complications and adverse clinical outcomes such as multiple organ failure, sepsis, neurological complications, and disability (6). MODS represents a critical state of multiorgan system failure, with a mortality rate as high as 60%; even if patients survive, many develop neurological impairments and limited skeletal muscle function, complicating patient management and treatment outcomes (7). Therefore, the prevention and management of MODS in patients with SHS is of paramount importance.

However, current research primarily focuses on treatment strategies for heat stroke and analyses of a single system or mortality risk factors, and there is a lack of studies on SHS complicated by MODS. Effective tools for assessing the risk of MODS in patients with SHS are currently unavailable. Acute Physiology and Chronic Health Evaluation II (APACHE II) and Sequential Organ Failure Assessment (SOFA) scores are the most widely used systems for evaluating the severity of critical illnesses. These scores have also been used in studies related to heat stroke to assess disease severity (8, 9). However, research has indicated that these scoring systems have limitations for diseases with specific characteristics, especially those involving distinct organ injuries or altered normal physiological parameters (9). The most common causes of MODS in SHS are coagulation disturbances including disseminated intravascular coagulation and acute kidney injury caused by heat damage and rhabdomyolysis. These factors are not adequately addressed in current scoring systems, highlighting the need for disease-specific scoring systems to accurately assess severity and predict outcomes. Given the rapid onset, progression, and high mortality rate of MODS in patients with SHS, accurate clinical risk assessment and proactive management strategies are crucial. This study aimed to analyze the risk factors for MODS in patients with SHS, develop and validate a prediction model, and provide assessment tools for the early identification, diagnosis, and treatment of MODS.

## 2 Methods

### 2.1 Study design

This study is a retrospective analysis involving 284 patients with SHS admitted to the ICU of two tertiary hospitals in China from July 2009 to April 2024. The study was approved by the ethics review committee, which waived the requirement for informed consent.

The inclusion criteria were as follows: (1) ICU patients diagnosed with SHS (ICD-10) in the electronic medical record system (2) no prior history of relevant conditions; (3) age ≥ 18 years. (4) SHS patients who did not receive organ support treatment within 24 h or whose condition could not be controlled and were transferred to our hospital. Patients were excluded for the following reasons: pregnancy or lactation, missing clinical data, transfer to another hospital, withdrawal from treatment or heat stroke secondary to trauma.

### 2.2 Definition of MODS

The definition of MODS was based on the consensus of the American College of Chest Physicians (ACCP) and the Society of Critical Care Medicine (SCCM) (10). MODS is defined as a clinical syndrome in which a patient without prior organ dysfunction experiences simultaneous or sequential dysfunction of two or more organs due to acute insults, such as severe trauma, infection, or shock, and cannot maintain homeostasis without intervention. In this study, the outcome indicator was the incidence of in-hospital MODS. Patients were evaluated and followed up according to the above definition. Based on the presence or absence of MODS during hospitalization, patients were divided into the MODS group and the non-MODS group.

### 2.3 Data collection

Based on an analysis of the literature related to SHS and MODS combined with clinical data and expert discussions, 34 potential predictors of MODS in patients with SHS were identified. These predictors included age, BT, heart rate (HR), respiratory rate (RR), acute gastrointestinal injury (AGI), Glasgow coma scale (GCS), mean arterial pressure (MAP), urine output (UO), blood glucose (BG) levels, white blood cell count (WBC), neutrophil percentage (Neu%), hemoglobin (Hb), hematocrit (HCT), platelet count (PLT), prothrombin time (PT), activated partial thromboplastin time (APTT), and serum levels of creatinine (Cr), alanine transaminase (ALT), aspartate transaminase (AST), chloride (Cl^−^), calcium (Ca^2+^), sodium (Na^+^), potassium (K^+^), creatine kinase (CK), lactate dehydrogenase (LDH), blood urea nitrogen (BUN), total bilirubin (TBil), creatine kinase-MB (CK-MB), fibrinogen (Fib), D-dimer (D-D), myoglobin (Mb), C-reactive protein (CRP), procalcitonin (PCT), and troponin I (Tn I). In addition, patients’ SOFA and APACHE II scores were collected for comparison of predictive efficacy. All data were obtained from the hospital’s electronic medical record system, with laboratory indicators taken from the first test results within 24 h of ICU admission. Two researchers collected the data and cross-verified their completeness, authenticity, and accuracy. Complete data records were managed by dedicated personnel. To minimize bias due to missing data, variables with more than 20% missing values were excluded from the final cohort and multiple imputation methods were used to complete the remaining data.

### 2.4 Statistical analysis

The study population was randomly divided into a training dataset (*n* = 198) and an internal validation dataset (*n* = 86) in a 7:3 ratio using the sample function in R. Data analysis was performed using SPSS Statistics version 26.0, whereas R version 4.2.0, was used to construct the nomogram, calibration curve, and decision curve analysis (DCA). Continuous variables with normal distribution were presented as mean ± standard deviation, while continuous data with a skewed distribution were expressed as median with interquartile range (IQR). Categorical variables were expressed as percentages (%). Pairwise comparisons were conducted using Student’s *t*-test or the Mann–Whitney *U* test for continuous variables, while the chi-squared test or Fisher’s exact test was used for categorical variables with normal or skewed distributions, as appropriate. Differences were considered significant if *p* < 0.05. Univariate logistic regression analysis was used to identify relevant risk factors, and variables with *p* < 0.05 were included in the multivariate logistic regression analysis to construct a risk prediction model for MODS in SHS patients, which was visualized using a nomogram.

The discriminative ability of the model was evaluated using a receiver operating characteristic (ROC) curve and the area under the curve (AUC). The Hosmer–Lemeshow (HL) test was used to verify the model’s goodness of fit, a calibration curve was used to assess prediction accuracy, and DCA was employed to evaluate the clinical utility of the model. The significance level of the above statistical analyses was set at *α* = 0.05 and *p* < 0.05 (two-tailed) for statistical significance.

## 3 Results

### 3.1 Baseline characteristics of patients

**Figure 1:**
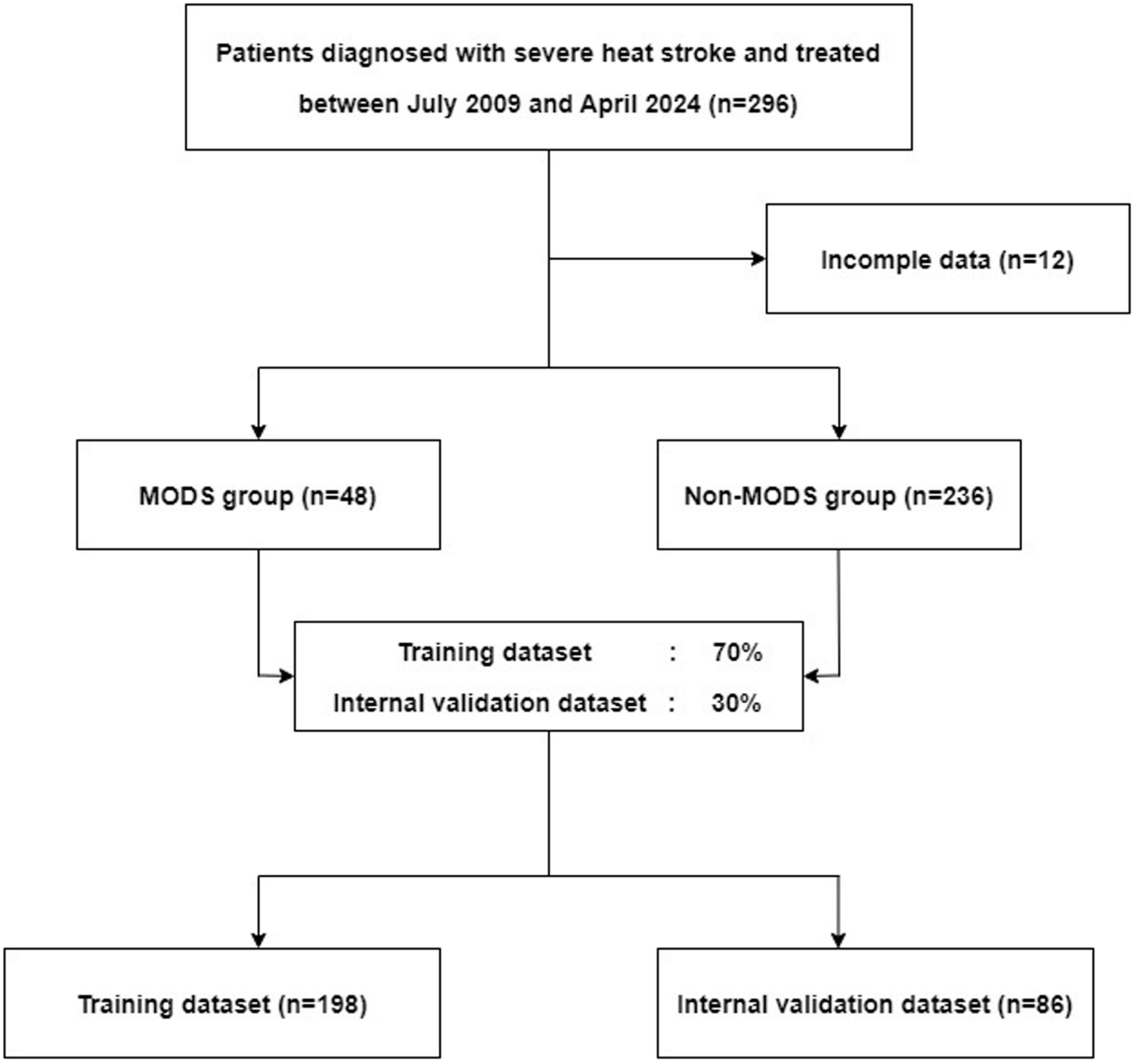
Flowchart diagram illustrating patients included in each group throughout the study.

Based on the inclusion and exclusion criteria, 284 patients were included in this study (Figure 1). The MODS and non-MODS groups included 48 and 236 patients, respectively. The average follow-up time for patients in this study was 22 days. The ages of the included patients ranged from 18 to 72 years, with a mean age of 26.3 ± 11.3 years. No significant differences were observed between the MODS and non-MODS groups in terms of Age, RR, MAP, BG, Cl^−^, Ca^2+^, Na^+^, K^+^, WBC, Fib, Mb, and CRP. The median BT in the MODS group was higher than in the non-MODS group (37.4°C vs. 36.9°C, *p* < 0.001), although still within the normal range. A significantly higher proportion of patients in the MODS group had HR >100 beats/min (50% vs. 8.5%, *p* < 0.001) and AGI (89.6% vs. 24.6%, *p* < 0.001). The mean GCS score in the non-MODS group was 3 points higher than that in the MODS group (*p* < 0.001). The median levels of ALT, AST, LDH, CK-MB, and D-D were significantly higher in the MODS group than in the non-MODS group (*p* < 0.001). The patient characteristics are presented in Table 1.

**Table 1:** Demographic and clinical characteristics of patients.

Characteristics	Overall (*n* = 284)	Non-MODS group (*n* = 236)	MODS group (*n* = 48)	*P*
Age, year	26.3 ± 11.3	25.5 ± 10.	30.4 ± 15.8	0.283
T, °C	37 (36.5, 37.6)	36.9 (36.5, 37.5)	37.4 (36.8, 38.3)	<0.001
HR, bpm				<0.001
>100	44 (15.5)	20 (8.5)	24 (50)	
≤100	240 (84.5)	216 (91.5)	24 (50)	
RR, bpm	19.9 ± 3.4	19.9 ± 2.9	20.2 ± 5.4	0.693
AGI				<0.001
Yes	91 (32)	58 (24.6)	43 (89.6)	
No	193 (68)	178 (75.4)	5 (10.4)	
GCS score	12 ± 4	13 ± 3	10 ± 4	<0.001
MAP, mmHg	85.6 ± 13.8	86.3 ± 13.2	82.2 ± 15.8	0.101
Cr, μmol/L	136.9 ± 94.4	120.6 ± 68.5	217.1 ± 149.4	<0.001
UO, ml/24 h	1966.9 ± 1,586	2073.7 ± 1,611	1,442 ± 1,353	0.012
ALT, U/L	53.5 (22, 325.8)	42.5 (20, 237)	225 (50, 835.5)	<0.001
AST, U/L	93.5 (35.3, 426)	77.5 (31, 297)	271.5 (88.5, 833.5)	<0.001
LDH, U/L	408 (260.3, 783.5)	388 (245, 783.5)	783.5 (379.3, 1,567)	<0.001
BG, mmol/L	6 ± 1.9	5.9 ± 1.8	6.4 ± 2.6	0.164
BUN, mmol/L	5.8 (4.4, 7.8)	5.4 (4.3, 7.3)	8 (5.5, 11.3)	<0.001
TBIL, μmol/L	16.5 (11.3, 32.6)	15.5 (11, 27.6)	32.7 (14.3, 67.2)	<0.001
CK, U/L	1,081 (405.8, 3196.9)	941.5 (337, 2915.5)	1,372 (679.5, 6471.5)	0.005
CK-MB, U/L	46 (23, 83.4)	39.5 (22, 83.4)	80 (35.3, 194.8)	<0.001
Cl^−^, mmol/L	105.5 ± 5.8	105.7 ± 5.6	104.6 ± 6.6	0.209
Na^+^, mmol/L	142.1 ± 5	141.8 ± 4.7	143.6 ± 6.3	0.078
Ca^2+^, mmol/L	2.1 ± 0.2	2.2 ± 0.2	2.1 ± 0.3	0.210
K^+^, mmol/L	3.7 ± 0.6	3.7 ± 0.5	3.8 ± 0.8	0.443
WBC, ×10^9^/L	11.2 ± 4.5	11.1 ± 4.4	11.9 ± 4.8	0.266
Neu%, %	83.2 (72.9, 88.7)	82.6 (72.4, 88.5)	86.6 (77, 91.3)	0.040
Hb, g/L	132.5 ± 21.3	135.4 ± 17.3	118.2 ± 31.4	<0.001
HCT, %	40 ± 6.4	40.8 ± 5.3	36.2 ± 9.2	0.002
PLT, ×10^9^/L	149 ± 84.6	159.7 ± 84.3	96.4 ± 64.7	<0.001
PT, s	16.1 (14.6, 20.2)	15.8 (14.4, 18.3)	20.6 (16.6, 33.5)	<0.001
APTT, s	55 ± 38.9	51.3 ± 36.1	73.1 ± 46.8	0.003
Fib, g/L	2.5 (2, 3)	2.5 (2, 3)	2.1 (1.3, 3.6)	0.092
D-D, mg/L	3 (0.6, 20)	2.5 (0.5, 15)	18.4 (2.4, 20)	<0.001
Mb, μg/L	862.7 ± 1700.7	823.4 ± 1824.1	1055.7 ± 856	0.389
CRP, mg/L	6.3 (3.2, 12.8)	6.4 (3.2, 12.8)	5.6 (3.2, 12.8)	0.750
PCT, ng/ml	2.1 (0.7, 4.9)	1.9 (0.5, 4.9)	3.3 (1.2, 7.7)	0.024
Tn I, μg/L	0.9 (0.2, 2.1)	0.7 (0.1, 1.9)	2 (0.5, 4.3)	<0.001

Values are *n* %, mean ± SD, or median interquartile range, unless otherwise noted. T, temperature; HR, heart rate; RR, respiratory rate; AGI, gastrointestinal injury; GCS, Glasgow coma scale; MAP, mean arterial pressure; UO, urine output; BG, blood glucose; WBC, white blood cell count; Neu%, neutrophil percentage; Hb, hemoglobin; HCT, hematocrit; PLT, platelet count; PT, prothrombin time; APTT, activated partial thromboplastin time; Cr, creatinine; ALT, alanine transaminase; AST, aspartate transaminase; Cl−, chloride; Ca^2+^, calcium; Na^+^, sodium; K^+^, potassium; CK, creatine kinase; LDH, lactate dehydrogenase; BUN, blood urea nitrogen; TBil, total bilirubin; CK-MB, creatine kinase-MB; Fib, fibrinogen; D-D, D-dimer; Mb, myoglobin; CRP, C-reactive protein; PCT, procalcitonin; Tn I, troponin I.

### 3.2 Univariate and multivariate logistic regression analysis of risk factors for MODS in SHS patients

Univariate logistic regression analysis identified BT, HR, AGI, GCS, Cr, UO, ALT, AST, LDH, and BUN as significant risk factors for the development of MODS in SHS patients (*p* < 0.05).

A multivariate logistic regression analysis was conducted to further investigate these associations. In the multivariate logistic regression model, MODS occurrence was set as the dependent variable (0 = non-MODS, 1 = MODS), and significant risk factors from the univariate analysis were included as independent variables. The results indicated that HR, AGI, GCS score, and TBil were independent risk factors for MODS in SHS patients (*p* < 0.05). The present study found that patients with SHS and HR >100 bpm were approximately eight times more likely to develop MODS than those with an HR ≤100 bpm. Additionally, SHS patients with AGI had seven times the odds of developing MODS compared to those without AGI injury. Furthermore, each 1-point decrease in the GCS score was associated with a 26% reduction in the probability of MODS. The results of the univariate and multivariate logistic regression analyses are presented in Table 2.

**Table 2:** Univariate and multivariate logistic regression analysis of risk factors for MODS in SHS patients.

Variables	Univariate logistic	*P*	Multivariate logistic	*P*
	OR (95%CI)		OR (95%CI)	
Age, year	1.010 (0.978–1.043)	0.561		
T, °C	1.632 (1.076–2.476)	**0.021**		
HR, bpm				
>100	10.286 (4.495–23.536)	**<0.001**	7.987 (1.722–37.036)	**0.008**
≤100				
RR, bpm	1.110 (0.907–1.359)	0.310		
AGI				
Yes	16.848 (6.125–46.339)	**<0.001**	7.213 (1.878–27.711)	**0.004**
No				
GCS score	0.753 (0.674–0.840)	**<0.001**	0.743 (0.628–0.878)	**<0.001**
MAP, mmHg	0.981 (0.954–1.008)	0.169		
Cr, μmol/L	1.010 (1.006–1.015)	**<0.001**		
UO, ml/24 h	1.000 (0.999–1.000)	**0.040**		
ALT, U/L	1.000 (1.000–1.001)	**0.036**		
AST, U/L	1.000 (1.000–1.000)	0.103		
LDH, U/L	1.000 (1.000–1.001)	**0.008**		
BG, mmol/L	1.062 (0.885–1.274)	0.517		
BUN, mmol/L	1.183 (1.078–1.298)	**<0.001**		
TBIL, μmol/L	1.012 (1.005–1.020)	**0.002**	1.018 (1.003–1.032)	**0.016**
CK, U/L	1.000 (1.000–1.000)	0.619		
CK-MB, U/L	1.003 (1.001–1.006)	**0.018**		
Cl^−^, mmol/L	0.970 (0.913–1.031)	0.328		
Na^+^, mmol/L	1.094 (1.009–1.186)	**0.029**		
Ca^2+^, mmol/L	0.567 (0.105–3.050)	0.508		
K^+^, mmol/L	1.700 (0.858–3.371)	0.129		
WBC, ×10^9^/L	0.990 (0.914–1.073)	0.811		
Neu%, %	1.011 (0.982–1.040)	0.468		
Hb, g/L	0.969 (0.952–0.985)	**<0.001**		
HCT, %	0.907 (0.857–0.960)	**<0.001**		
PLT, ×10^9^/L	0.989 (0.983–0.995)	**<0.001**		
PT, s	1.034 (1.007–1.062)	**0.014**		
APTT, s	1.011 (1.003–1.018)	**0.004**		
Fib, g/L	0.731 (0.478–1.118)	0.148		
D-D, mg/L	1.000 (1.000–1.000)	0.273		
Mb, μg/L	1.001 (1.000–1.001)	**0.016**		
CRP, mg/L	1.009 (0.997–1.021)	0.151		
PCT, ng/ml	1.027 (0.999–1.056)	0.058		
Tn I, μg/L	1.251 (1.082–1.446)	**0.002**		

Values in bold are statistically significant variables. MODS, multiple organ dysfunction syndrome; SHS, severe heat stroke.

### 3.3 Development of the predictive model

**Figure 2:**
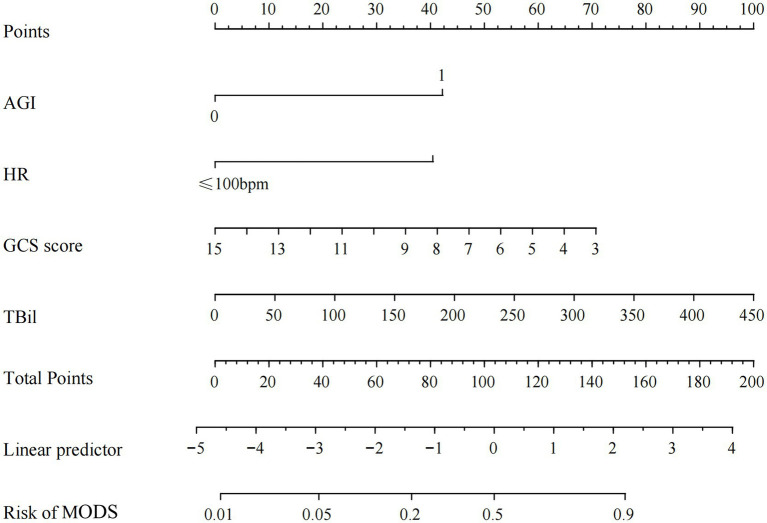
Nomogram for predicting secondary MODS in patients with severe heat stroke.

A risk prediction model for MODS in patients with SHS was developed based on binary logistic regression analysis. The model was constructed by extracting the regression coefficients of the identified risk factors and fitting a logistic regression equation to predict the probability of developing MODS. Based on this model, a nomogram was created to visualize the predictive model (Figure 2).

The model equation was as follows: Logit (P) = 0.305 + 1.976 × AGI + 2.078 × heart rate (>100 bpm) − 0.297 × GCS + 0.018 × total bilirubin; P means the probability of SHS patients developing MODS.

### 3.4 Validation of the predictive model

#### 3.4.1 Goodness of fit test

**Figure 3:**
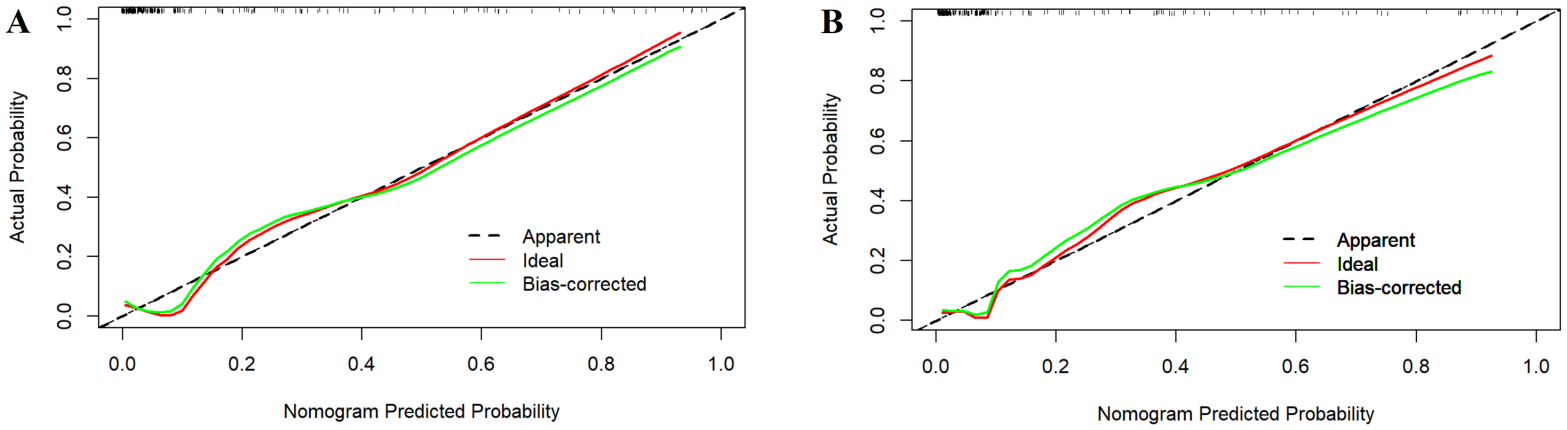
Calibration curves for the training dataset **(A)** and internal validation dataset **(B)**.

The goodness-of-fit of the risk prediction model was verified using the HL test. The HL test results indicated that the *p*-values for the training and internal validation datasets were 0.234 and 0.978, respectively (*p* > 0.05). This demonstrates that the probabilities predicted by the MODS risk-prediction model were consistent with the observed occurrences, indicating a good fit. The calibration curves for the training and internal validation datasets closely aligned with the ideal line, suggesting that the predicted and actual results were in good agreement (Figure 3).

#### 3.4.2 Discriminant validity test

**Figure 4:**
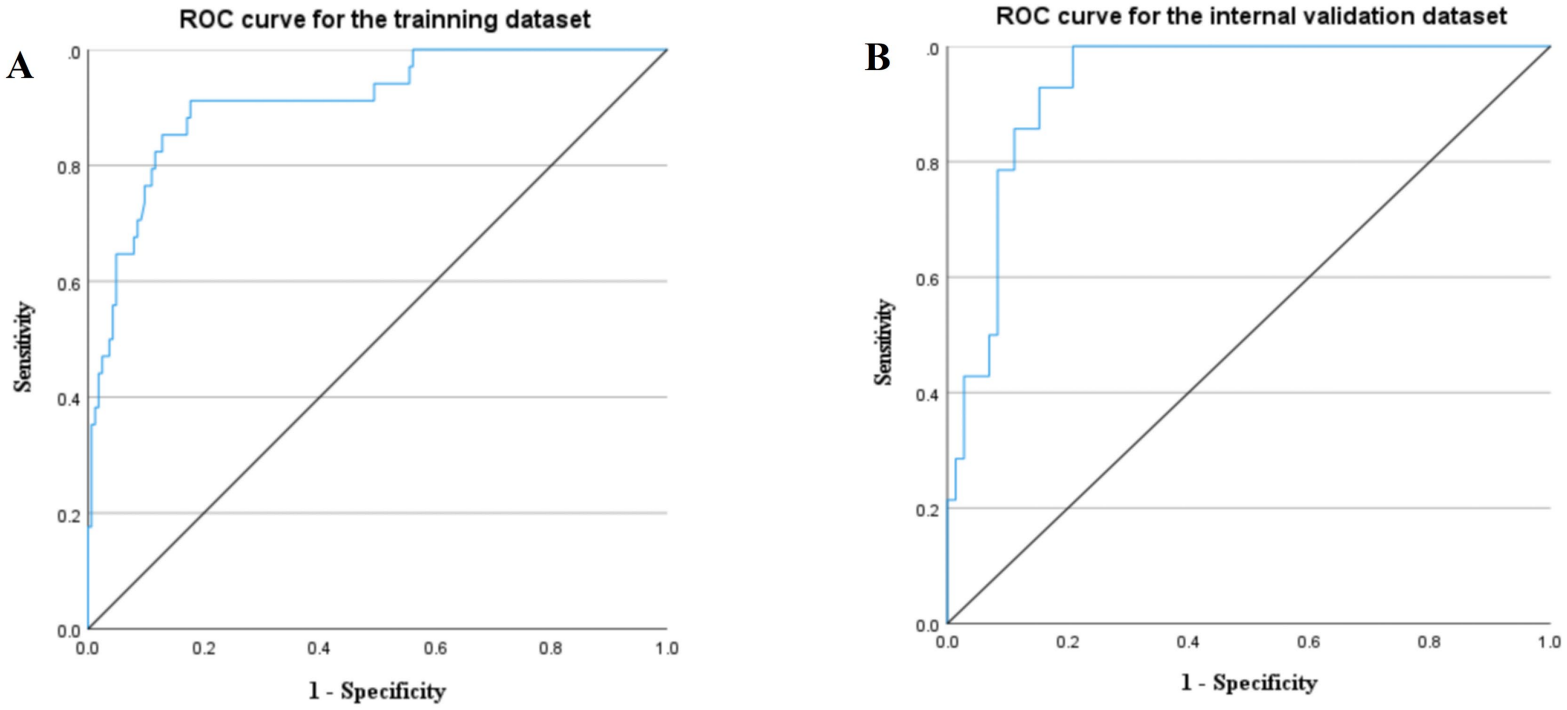
Receiver operating characteristic (ROC) curve for the training dataset **(A)** and internal validation dataset **(B)**.

The area under the curve (AUC) was calculated by plotting the ROC to assess the discriminatory ability of the model. The AUC values for the training and internal validation datasets were 0.910 (95% CI: 0.856–0.965) and 0.933 (95% CI: 0.880–0.985), respectively, indicating that the model effectively distinguishes between patients with and without MODS in SHS (Figure 4). The sensitivity (Sen) and specificity (Spe) of the model, determined by the optimal Youden index (YI) cutoff point on the ROC curve, were 0.912 and 0.823 (with a Youden index of 0.735 and optimal cutoff value of 0.123) for the training set, and 1.000 and 0.792 (with a Youden index of 0.792 and optimal cutoff value of 0.309) for the internal validation dataset.

**Figure 5:**
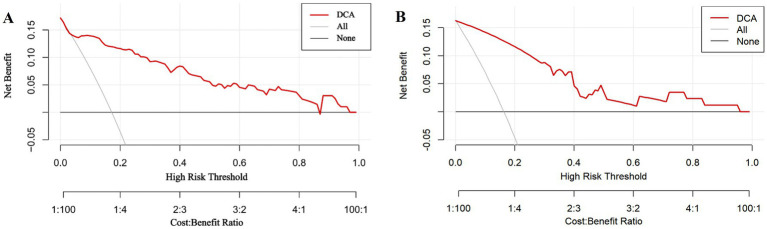
Decision curve analysis (DCA) results for the training dataset **(A)** and internal validation dataset **(B)**.

The DCA showed that the decision curves for both the training and internal validation datasets were far from the baseline, indicating that the prediction model had a substantial net clinical benefit (Figure 5).

#### 3.4.3 Comparison of predictive models, SOFA scores, and APACHE II scores

**Figure 6:**
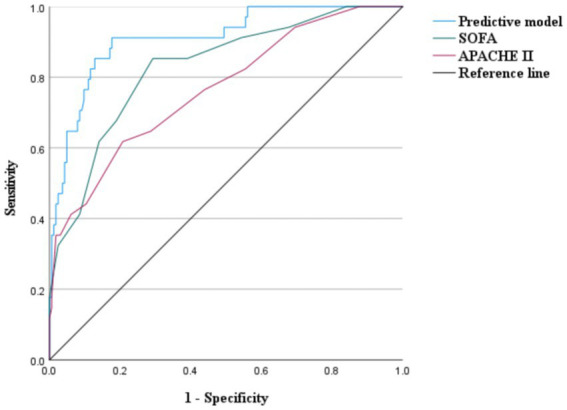
Receiver operating characteristic for the predictive model, SOFA score, and APACHE II score.

When comparing the prediction model with the SOFA and APACHE II scores, the AUC, sensitivity, and specificity of the predictive model (0.910, 0.912, and 0.823, respectively) were significantly higher than those of both the SOFA (0.828, 0.618, and 0,793) and APACHE II scores (0.766, 0.853, and 0.707), demonstrating superior predictive performance (Figure 6 and Table 3).

**Table 3:** Comparison of predictive models, SOFA Scores, and APACHE II Scores.

Test result variables	AUC	95%*CI*	*P*	Cutoff	Sen	Spe	YI
Predictive model	0.910	0.856–0.965	<0.001	0.123	0.912	0.823	0.735
SOFA	0.828	0.751–0.904	<0.001	7.50	0.618	0.793	0.410
APACHE II	0.766	0.675–0.857	<0.001	5.50	0.853	0.707	0.560

SOFA, sequential organ failure assessment; APACHE II, acute physiology and chronic health evaluation II; AUC, area under the curve; Sen, sensitivity; Spe, specificity; YI, Youden index.

## 4 Discussion

Shock, acute renal failure, rhabdomyolysis, acute respiratory distress syndrome, disseminated intravascular coagulation, and acid–base or electrolyte imbalances are common complications of severe heat stroke (SHS) (3). Once these conditions progress to MODS, the mortality rates and risk of adverse outcomes increase significantly (11), underscoring the importance of developing predictive models for MODS in SHS. This study found that AGI, HR > 100 bpm, a decreased GCS, and elevated TBil within the first 24 h of ICU admission are independently associated with the development of MODS in patients with SHS and can serve as risk factors for identifying high-risk individuals.

Our study found that AGI was a significant independent risk factor for MODS in patients with SHS, which is consistent with the findings of previous research (12). The gastrointestinal tract is one of the primary organs affected during SHS owing to its high metabolic rate and extensive microvascular network, making it particularly susceptible to heat stress. The early manifestations of AGI, including gastrointestinal dysfunction, can occur during the initial stages of the disease. Research has demonstrated that the exacerbation of gastrointestinal injury is significantly associated with poor outcomes in critically ill patients. Stress-induced gastrointestinal bleeding can result in mortality rates as high as 40%, which is substantially higher than the mortality rates due to failure of other organs (13). Carrico et al. (14) first proposed the “gut-origin theory” of MODS in 1986, suggesting that the gastrointestinal tract initiates a gut-derived secondary hit. The first hit occurs when heat stress disrupts the tight junctions of epithelial cells, damages the cell membrane, and increases the permeability of the intestinal mucosa. The second hit involves a reduction in blood flow to the sacroiliac region, leading to intestinal ischemia and hypoxia. This results in local acidosis, altered ion pump activity, and the generation of reactive oxygen and nitrogen species. Together, the two hits compromise the intestinal mucosal barrier and increase intestinal permeability, facilitating the translocation of endotoxins and lipopolysaccharide (LPS) into the bloodstream. A previous study (15) showed that elevated LPS levels in the blood cause a further systemic inflammatory response. When LPS binds to Toll-like receptor 4 (TLR4) on immune cells, it can activate a variety of inflammatory pathways, including the release of cytokines (such as TNF-αand IL-6), resulting in a cytokine cascade. Inflammatory cascades can lead to capillary leakage, organ dysfunction, and gastrointestinal injury (16). As early as the last century, Bouchama et al. (17) indicated that hyperthermia and endogenous endotoxemia pathways create secondary stress, leading to uncontrolled inflammatory responses and complications, such as MODS. Our study findings align with these perspectives, showing that the AGI incidence in patients with SHS was 32.04%, with significantly higher AGI rates in the MODS group than in the non-MODS group (89.6% vs. 24.6%, *P* < 0.001). Therefore, early identification and management of AGI are crucial because controlling gastrointestinal dysfunction can prevent the development of MODS and improve patient outcomes.

HR > 100 bpm was also a significant prognostic indicator of MODS in patients with SHS, which is consistent with previous studies (18). Tachycardia, defined as an HR exceeding 100 beats/min, is a common physiological response to hyperthermia. The relationship between tachycardia and MODS during heat stroke is complex. BT directly affects HR, with heat stress increasing HR by approximately 40% owing to elevated cardiac BT, whereas the remaining 60% increase is mediated by the autonomic nervous system (19). Prolonged elevated BT triggers a reflexive increase in HR to maintain adequate arterial pressure, increases myocardial oxygen consumption, and potentially leads to myocardial infarction (20). Persistently elevated HR serves as a marker of cardiovascular stress, with prolonged tachycardia increasing the myocardial oxygen demand (21). Heat stroke can result in myocardial ischemia and subsequent heart failure, particularly in patients with pre-existing cardiovascular conditions or heat-induced myocardial injury. Additionally, tachycardia can indicate systemic inflammation and sepsis, both of which are crucial in the pathogenesis of heat stroke. The release of inflammatory mediators such as catecholamines, cortisol, and cytokines stimulates the autonomic nervous system, causing increased HR (22). Thus, in the absence of hypovolemia or fever, persistent tachycardia can be an early indicator of systemic inflammatory response and impending organ dysfunction.

A low GCS score at admission is a significant predictor of poor outcomes and MODS development in patients with SHS, which is consistent with recent studies (23, 24). Our findings revealed that the mean GCS score in the MODS group was lower than that in the non-MODS group (9.50 vs. 12.76; *p* < 0.001). Previous studies have shown that GCS score is significantly associated with in-hospital mortality in SHS patients (25), GCS score is an independent risk factor for 90-day mortality in exertional heat stroke (EHS) patients (26), and GCS < 12 is an independent prognostic factor for mortality in classic heat stroke (CHS) patients (27). An altered mental status is a hallmark of SHS, reflecting the significant involvement of the central nervous system and potential irreversible neuronal damage. Lower GCS scores are associated with a higher likelihood of central nervous system sequelae (28). However, the mechanisms by which heat stroke induces central nervous system dysfunction and MODS are complex and multifactorial. Studies have shown that brain cells are highly sensitive to elevated BT, with hyperthermia causing direct thermal injury to brain cells, protein denaturation, cell membrane disruption, and neuronal death (29), leading to central nervous system dysfunction. Heat stroke is associated with cerebral edema, hemorrhage, and microthrombosis, with hyperthermia-induced blood–brain barrier disruption allowing inflammatory and neurotoxic substances to enter the brain, further impairing cerebral perfusion and oxygenation and altering microcirculatory blood flow, ultimately progressing to MODS (30). The severity of central nervous system dysfunction not only indicates the extent of brain injury but also reflects the severity of MODS and the risk of adverse outcomes. Furthermore, BT admissions are closely related to the risk of central nervous system sequelae. Patients with SHS, higher BT on admission, and lower GCS scores were more likely to develop central nervous system sequelae, requiring longer cooling times to reach the target BT (31). Additionally, patients with SHS and significant central nervous system damage are more likely to develop hypothermia, with the lowest observed BT within 24 h significantly correlated with neurological sequelae (32). Therefore, immediate and aggressive cooling is crucial for mitigating central nervous system dysfunction and MODS.

Total bilirubin is a marker of liver function and has been identified as an independent risk factor of MODS in patients with SHS, which is consistent with previous findings (12). TBil level was also used as an indicator of liver injury based on the SOFA score. The liver is considered one of the earliest organs affected by MODS due to SHS, and acute liver injury is a common complication that can progress to acute liver failure in severe cases (33). It has been previously shown in animal studies (34, 35) that inflammatory cytokines IL-1β and IL-6 are released in response to heat stress and intestinal translocation of LPS. The two synergistically amplify the inflammatory response of heat stroke, forming a feedback loop in which more immune cells are recruited and more inflammatory cytokines are released. This worsens liver injury and contributes to systemic inflammation that affects other organs. Heat stroke-induced liver injury is characterized by elevated AST levels, which correlates with the degree of hepatocyte damage (36). Unlike AST, ALT and TBil levels rise approximately 2 days later and remained elevated for several days. Our study showed that AST levels were significantly higher than ALT levels (93.5 vs. 53.5), with TBil levels within the normal range (16.5, *p* < 0.001), consistent with previous findings. The median TBil value in the MODS group exceeded the normal range by nearly two-fold, indicating impaired liver clearance, which can lead to systemic toxicity. In patients with heat stroke, prolonged TBil elevation is correlated with the severity of liver damage (37). When TBil levels remain elevated, hyperbilirubinemia can develop, resulting in liver dysfunction, bile duct obstruction, excessive red blood cell destruction, jaundice, coagulopathy, and hepatic encephalopathy (38). TBil itself has pro-inflammatory properties, exacerbating systemic inflammation and oxidative stress, creating a vicious cycle and promoting MODS progression. Notably, although the liver is considered a sentinel organ in SHS, its strong compensatory mechanisms can delay the detection of liver injury, which is typically identifiable only at 24 h post-onset (39). However, a study found that 60.53% of patients could exhibited detectable liver injury within 24 h of admission, primarily presenting with simultaneous elevations in ALT, AST, and TBil, predominantly mild (40). Our study included indices obtained within 24 h of admission, emphasizing the need to closely monitor TBil levels to prevent ongoing liver damage and potential MODS progression.

Univariate analysis in this study found that a higher admission BT was associated with MODS occurrence; however, multivariate results indicated that it was not a definitive predictor of MODS. Initial hyperthermia in SHS is a major contributor to the damage, making BT a critical clinical parameter. The higher the core BT, the more severe is the damage to cell membranes and intracellular structures, resulting in a more severe systemic inflammatory response, ultimately leading to MODS and death. However, studies have pointed out that the duration of hyperthermia, rather than its intensity, is associated with poor outcomes (41, 42). According to the 2020 Chinese Expert Consensus on Standardized Diagnosis and Treatment of Heat Stroke, rapidly reducing core BT to below 39°C within 40 min or below 38.5°C within 2 h is considered crucial in managing SHS patients. Clinically, core BT guides cooling therapy; core BT refers to the “cavity” (thoracic and abdominal) BT compared to surface BT. Because direct measurement of the thoracoabdominal BT is not feasible, ear BT is commonly used as a substitute. In this study, ear BT was performed instead of core BT. The lack of data on the duration of hyperthermia and the fact that most patients underwent cooling treatment upon admission may have led to normal BT readings on admission, reducing the statistical significance of BT in the predictive model.

APACHE II and SOFA scores are widely used in clinical practice, particularly in critically ill patients. Higher scores indicated more severe physiological disturbances and a greater likelihood of developing MODS. A study indicated that the SOFA score can better predict mortality in patients with heat-related illnesses than other scoring systems, with an AUC of 0.83 (43). The SOFA scores in our study were similar (AUC, 0.828; 95% *CI*: 0.751–0.904, *p* < 0.001). In this study, the AUC of APACHE II scores was lower than that of the predictive model and SOFA score, possibly because early SHS can cause liver and coagulation dysfunction, which was not assessed using APACHE II, which is consistent with previous findings (44). The AUC, sensitivity, and specificity of the MODS predictive model constructed in this study were higher than those of the SOFA and APACHE II scores (*p* < 0.001), indicating the superior predictive performance of the model.

This study developed a risk prediction model for MODS in patients with SHS based on binary logistic regression analysis. The model demonstrated a good predictive performance, with AUC values for the training and internal validation datasets exceeding those of the SOFA and APACHE II scores. The calibration curve and DCA indicated a good predictive accuracy and clinical utility. Visualization of the model through a nomogram aids clinicians in assessing MODS risk and implementing targeted interventions to reduce the incidence of MODS.

However, this study has some limitations. First, this was a retrospective study, necessitating further validation in prospective cohorts and clinical trials. Secondly, data on the duration of illness and hospital admission were not included in this study, so there are potential differences in the time from heat stroke onset to ICU admission. This study also lacked additional biomarkers associated with heat stroke, such as IL-6, LPS, BNP, FDP, μMb, and BE. The limitations of GCS due to confounding factors such as medications, sedatives, and other medications and its unreliability in some populations (e.g., those with disabilities and cognitive impairments) have not been addressed. Finally, the model was based on laboratory indicators obtained within the first 24 h of admission, limiting its predictive scope to the initial phase of illness and not reflecting the entire clinical course. We will conduct a longitudinal study to validate and refine the model’s effectiveness in predicting the risk of secondary MODS during hospitalization in patients with SHS.

## 5 Conclusion

Patients with SHS who develop MODS have high mortality rates. Early assessment and intervention of MODS risk in these patients are crucial for reducing the incidence and mortality associated with MODS. This study identified AGI, HR > 100 bpm, a decreased GCS, and elevated TBil within the first 24 h of ICU admission are identified as independent risk factors for MODS in patients with SHS. The risk prediction model for MODS developed in this study demonstrated excellent predictive performance and clinical utility, and can serve as an objective and convenient tool. By measuring the score of each item, the patient’s risk degree of MODS and high-risk affected organs were identified. For SHS patients with high-risk AGI, fluid resuscitation and selective digestive decontamination can be initiated at an early stage to reduce the risk of bacterial translocation and infection. Patients with heart rate > 100 bpm can timely strengthen vascular support and optimize fluid management to prevent circulatory failure and organ hypoperfusion, so as to reduce the risk of MODS. Elevated total bilirubin suggests liver dysfunction. Hepatoprotective strategies can be adopted in patients with hyperbilirubinemia, including careful management of hepatotoxic medications and liver support therapy to avoid the cascading effects of multiorgan involvement. For high-risk patients with lower GCS score, monitoring should be strengthened. Neuroprotective strategies, such as maintaining optimal oxygenation and intracranial pressure management, should be adopted as early as possible to reduce the risk of brain injury progressing to MODS. By integrating these predictive model-based clinical interventions, health care providers may be able to stabilize high-risk SHS patients earlier when they are admitted to the ICU, slowing their progression to MODS, and thereby reducing mortality. The model provides a structured, indicator-based approach for clinicians to proactively manage patients with heat stroke, ideally leading to more efficient allocation of ICU resources and improved patient outcomes.

## Data Availability

The data that support the findings of this study are available on request from the corresponding author. The data are not publicly available due to privacy or ethical restrictions.
